# Experiments and Identification of the Unbalance of Aerostatic Guideways on the Micro-Scale

**DOI:** 10.3390/s140304416

**Published:** 2014-03-05

**Authors:** Dongju Chen, Yanhua Bian, Jinwei Fan

**Affiliations:** College of Mechanical Engineering & Applied Electronics Technology, Beijing University of Technology, Beijing 100124, China; E-Mails: bianyanhua@emails.bjut.edu.cn (Y.B.); jwfan@bjut.edu.cn (J.F.)

**Keywords:** unbalance, gas fluctuation, aerostatic guideway, micro-scale, identification

## Abstract

In order to diagnose the unbalance of aerostatic guideways, displacement and acceleration sensors were used to measure the vibration signals of the unbalance of the aerostatic guideways caused by the gas fluctuation. The frequency characteristics for the unbalance of aerostatic guideways caused by gas fluctuation has been extracted from the measured signals by power spectral density, and the basic frequencies of the guideway system have been diagnosed according to spectral characteristics, in agreement with the results calculated by the corresponding motion equations and the finite element method.

## 1. Introduction

Due to their low friction and high accuracy of their motion aerostatic bearings and guideways have been successfully applied to various precision devices such as precision machine tools, precision measuring equipment and lithography associated production equipment [1]. Those advantages of aerostatic guideways cannot however always be realized to the full due to some limitations, in particular, their low specific stiffness and their liability to develop negative damping, which may lead to a type of self-excited vibration known as “pneumatic hammer” [2], under certain working conditions, whose likelihood seems to increase the more one tries to enhance the static characteristics [3]. In other words, dynamic stability represents an additional limit-line for bearing design optimization. However, in understanding the problem of dynamic stability, we hope not only to be able to predict its likelihood, in order to avoid it, but eventually to also be able to overcome it by modifying either the bearings or the supporting structures or both.

Currently, positioning error analysis [4] for such machines focuses on the relationship between volumetric errors on the one hand and axes' motion errors on the other. The internal mechanisms causing motion errors are rarely considered. Problems with conventional linear aerostatic guideways for an ultra-precision machine tool include deviation from straightness or parallelism tolerance between the granite guideways, and though the aerostatic slideway is stable at the equilibrium point, a sudden small displacement may cause a disturbance. Thus, the optimal air film gap will be changed, resulting in vibrations and affecting the positioning accuracy for an ultra-precision machine tool. The key to utilizing all these aspects and possibilities is a good fundamental understanding of the dynamic behavior of air-bearing films. This is the main subject to this paper, although there has been much work on the static performance of air bearings [5,6]. In 1999 the dynamic tilt characteristics of the rectangular aerostatic thrust bearing in the rolling and yaw direction with compound restrictors has been studied [7], The authors proposed that the capacity and stiffness of the aerostatic thrust bearing are high, but it is easy to produce pneumatic hammer instability [8]. Hua *et al.* [9] researched the effect of the environment humidity and temperature on the flying height of an air bearing slider, and its transient flying response was considered. Chen *et al.* [10] proposed that there exists a large vortex in the recess of bearings with spherical or rectangular recesses, and this vortex can cause the temperature in the film to be higher than the ambient one, which may affect the stability of the bearing. Subsequently, Aoyama *et al.* [11] analyzed the behavior of the air flow in the bearing clearance of aerostatic guideways with the “Star-CD” software, and the mechanism of the small vibrations of aerostatic guideways was suggested and tested experimentally. Chen *et al.* [1] established the relationship between the gas vortices and nano-vibrations in aerostatic bearings. Zhu *et al.* [12] computed the pressure fluctuation caused by the vortex shedding in the bearing clearance, and established a relationship between the pressure fluctuation and bearing vibration.

In 1994, Zhao *et al.* [13] investigated the nonlinear dynamic behavior of an eccentric squeeze film damper-mounted rigid rotor system. The authors showed that for large values of unbalance and static misalignment, the sub-harmonic and quasi-periodic motions generated at speeds of more than twice the system critical speed were bifurcated from the unbalance harmonic solution. Czolczynski and Kapitaniak [14] described a method which allows the control of the Hopf bifurcation of a rotor system supported by two gas bearings. They showed the damage caused by the growing amplitude of self- excited vibration, and that this phenomenon can be avoided by a proper selection of stiffness and damping coefficients of the air ring.

Although previous works provide insights into the static and dynamic behaviors of the aerostatic guideway, they seldom mention the gas fluctuations or the hammer phenomenon of the aerostatic slider. In a vertical turning machine tool, gas fluctuation of the cross aerostatic slider of the machine tool will greatly affect the machining quality, but the reasons behind the gas film fluctuation of aerostatic guideways with mass unbalance have not been researched. Therefore, this paper researches the gas film fluctuation of aerostatic guideways with mass unbalance.

Due to the nonlinearity of the gas film pressure, it is very difficult to solve the Reynolds equation analytically. Therefore, motion equations of the aerostatic guideways and finite element methods have been employed to obtain the solution. In order to extract and identify the gas fluctuations of the aerostatic guideways, this paper investigates the relationship between the dynamic characteristic of the aerostatic guideways and the gas fluctuation both mathematically and experimentally, and the main features of the film fluctuation are extracted. The unbalance of aerostatic guideways caused by the fluctuation is identified in the frequency domain.

## 2. Experimental Section Analysis for the Dynamic Characteristic of the Aerostatic Guideway System

**Figure 1.**
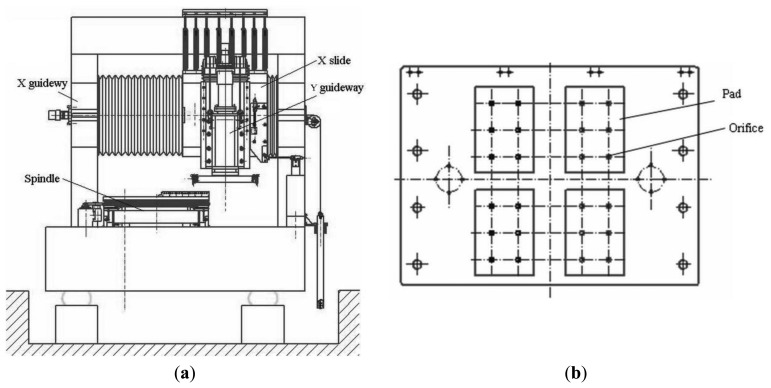
(**a**) Configuration of the guideway; (**b**) Structure of the aerostatic slider.

Aerostatic bearings support objects using air pressure in a non-contact state and thus enable ultra-precise motion of the object without friction. Their life is almost infinite and using these bearings reduces the environmental load because they only consume air, and not lubrication oils. Aerostatic bearings have restriction mechanisms to achieve a high level of stiffness. An aerostatic guideway with an orifice restriction in the laboratory is researched. The guideway is mounted on a granite base, with four air pads, and each pad has six orifices, the restriction type of the X slider is an orifice restriction, and the frame as shown in Figure 1. Figure 1a shows the total configuration of a vertical turning machine, including two guideways in the *x* and *y* directions, respectively, where the X guideway is the aerostatic guideway. The inner structure of the slider of X guideway is shown in Figure 1b.

For a vertical machine tool, when turning a workpiece, there is a slight fluctuation of the gas film between the cross slider and guideway, and a forced vibration in the slider will be caused by the self-excited oscillation of the gas film fluctuation. In order to study the effect of the vibration on the machining accuracy, the dynamic behavior of the aerostatic guideway system is next analyzed by motion equations and the finite element method, respectively.

### 2.1. Analysis by the Motion Equations

The guideway system will be subject to periodic excitation during the machining process. Resonance will occur when an excitation frequency coincides with one of the modal frequencies of the guideway, which will not only reduce the quality of the machined workpiece, but also potentially damage the tool and machine tool. Here the frequency information of the guideway system is analyzed with the transfer matrix method [15].

Modal analysis is used to understand the natural frequency in order to predict, evaluate and optimize the vibration behavior [16]. The primary deformation of the cross guideway is bending when its vibration is perpendicular to the axis line, which is a cross vibration of the guideway. Here, only the cross vibration of the guideway in *x*-*y* plane (the vertical plane in Figure 1a) is considered. Since the cross-sectional dimensions of the guideway is much smaller than its length, the guideway is simplified into a beam model, and the variation of the inertia and shear deformation are very small in the motion of the guideway, *y* = *y*(*x*, *t*), therefore, all the loads act on this plane. The guideway may be seen as a beam with both ends fixed, according to the cross vibration equation [17]:

(1) $$\frac{\partial^{4}y}{\partial x^{4}}=-\frac{1}{c^{2}}\frac{\partial^{2}y}{\partial t^{2}}$$

where, $c=\sqrt{\frac{EI}{m}}$, EI is the bending stiffness of the beam, *m* is the beam mass per unit length. The boundary conditions at which the deflection and rotation angles are null at both ends of the guideway are considered to solve the above equation, a*n*d the fifth-order frequencies are obtained and shown in Table 1.

### 2.2. Analysis by the Finite Element Method

**Figure 2.**
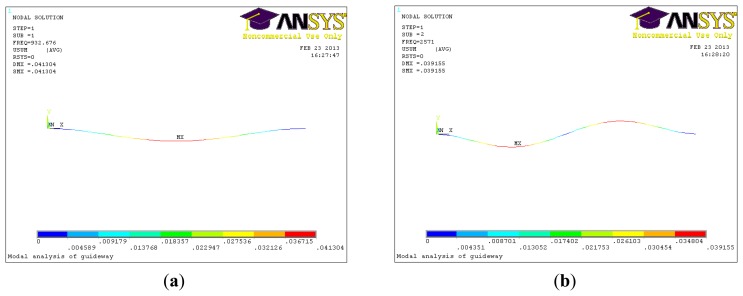
(**a**) Vibration shape of first order; (**b**) Vibration shape of second order.

The finite element method (FEM) was used in order to verify the validity of the above method. First, a three-dimensional CAD model is established and imported into the ANSYS software. After modeling, the necessary data of the material properties of guideway are assigned to the beam element (Young's modulus 2.0 × 10^9^ N/m^2^, Poisson's ratio 0.3 and mass density 8.58 × 10^3^ kg/m^3^). The natural frequencies are shown in Table 1, it shows that the natural frequencies of the guideway obtained by the analytical theory and ANSYS are very close. Figure 2 shows the simulated results of the modal shape and frequency, Figure 2a is for the first-order, showing bending only in one direction, and Figure 2b is for the second-order, showing bending in two directions.

### 2.3. Gas Film Analysis Based on Slip Velocity Method

In order to identify the causes of the gas fluctuation of the guideways, we focused on the airflow in the clearance of the gas pads because the pressure fluctuation due to unsteady airflow may induce the fluctuation. Since the thickness of the gas film of the aerostatic guideway is 10 μm, it corresponds to the micro-scale, therefore, the flow state of the gas film of the guideway is also on the micro-scale. The Navier velocity slip boundary conditions are adopted to the fluid continuous flow equation and the Reynolds equation is modified in the micro-scale. Based on the conservation of oil film flow and the linearization assumption of the oil film pressure distribution, the load capacity of the bearing is gained in the consideration of slip effect. The Reynolds equation for a compressed gas based on the Navier-Stokes equations is used to identify the behavior of the airflow. The basic equation of pressure distribution in the gas film is [18]:

(2) $$\frac{\partial }{\partial x}(ph^{3}\frac{\partial p}{\partial x})+\frac{\partial }{\partial y}(ph^{3}\frac{\partial p}{\partial y})=12\eta \frac{\partial (ph)}{\partial t}+6\eta u\frac{\partial (ph)}{\partial x}+6\eta v\frac{\partial (ph)}{\partial y}$$

where *p* is the pressure of the gas film, and *h* is the film thickness, *η* is the dynamic viscosity of the air, *u*, *v* and *w* are the velocity of the gas flow in *x*, *y* and *z* direction respectively, *v* is much smaller than *u* and *w*, it can be seen zero. *t* is the time parameter. The left part of the equation is the film pressure distribution along the *x*, *z* direction, and it is a two-dimensional relationship. The *y* direction is consistent with the thickness of the gas film, and the *x*-*z* plane is in contact with the gas film surface. The first part on the right of the Equation (2) expresses the squeeze film role caused by the relative speed of the two gas lubrication surfaces in the normal direction. The second and third ones on the right of the Equation (2) are the speed of the movement or the rotation of the lubricated motion surfaces. For the analysis, several assumptions are made:

- There is no heat exchange in the gas film of the whole process, and the temperature is constant.
- The fluid in the gas pad is a Newtonian fluid.
- The effect of inertia force on the fluid is ignored (compared with damping and gravity, it is very small).
- The pressure variation along the thickness direction of the film is zero.

The momentum equations in *x* and *y* direction are $\frac{\partial p}{\partial x}=\eta \frac{\partial^{2}u}{\partial y^{2}}$, $\frac{\partial p}{\partial z}=\eta \frac{\partial^{2}w}{\partial y^{2}}$, $\frac{\partial p}{\partial y}=0$, state equation of gas film *P* = *ρ* · *R* · *T*, and continuity equation $\frac{\partial \left(\rho u\right)}{\partial x}+\frac{\partial \left(\rho v\right)}{\partial y}+\frac{\partial \left(\rho w\right)}{\partial z}+\frac{\partial \rho }{\partial t}=0$.

The boundary conditions for the velocity slip are different from conventions, and are:

(3) $$\begin{array}{cccc} y=0, & u=\text{U}+l^{'}\;\frac{\partial u}{\partial y}, & w=l^{'}\;\frac{\partial w}{\partial y}, & v=0 \end{array}$$

(4) $$\begin{array}{cccc} y=h, & u=\text{U}-l^{'}\;\frac{\partial u}{\partial y}, & w=-l^{'}\;\frac{\partial w}{\partial y}, & v=0 \end{array}$$

where $l^{'}=\frac{2-\sigma_{v}}{\sigma_{v}}\cdot \lambda$, is the slip length of the fluid, λ is the average free path of gas molecules, *σ_v_* is the tangential momentum adjustment coefficient of the molecule, which means that the molecular proportion of the diffuse reflection occurs in the object surface. It is associated with the nature of the fluid medium and the surface roughness. Through the above equations, the Reynolds equation is derived:

(5) $$\frac{\partial }{\partial x}(ph^{3}\frac{\partial p}{\partial x})+\frac{\partial }{\partial y}(ph^{3}\frac{\partial p}{\partial y})=\frac{6\mu U}{1+6k_{n}^{\prime }}\times \frac{\partial (ph)}{\partial x}+\frac{12\mu }{1+6k_{n}^{\prime }}\times \frac{\partial \left(ph\right)}{\partial t}$$

where $k_{n}^{\prime }=\frac{2-\sigma_{v}}{\sigma_{v}}\cdot k_{n}$, *U* is the speed of slide plate. The dimensionless form of the above equation is made, where *p*_0_, *h_m_*, *l*, *V* are the reference quantities, *p*_0_ is gas pressure, *h_m_* is the gas film thickness, *l* is the selected reference length, *V* is the motion velocity. By assuming *p*=*p*_0_·*p̅*, *h*=*h_m_*·*h̅*, *x*=*l*·*x̅*, *y*=*l*·*y̅*, *t*=(*l*/*V*)·*t̅* and according to the equation $p\frac{\partial p}{\partial x}=\frac{1}{2}\cdot \frac{\partial p^{2}}{\partial x}$, $p\frac{\partial p}{\partial y}=\frac{1}{2}\cdot \frac{\partial p^{2}}{\partial y}$, Equation (5) becomes:

(6) $$\frac{\partial }{\partial \bar{x}}({\bar{h}}^{3}\frac{\partial p^{2}}{\partial \bar{x}})+\frac{\partial }{\partial \bar{y}}({\bar{h}}^{3}\frac{\partial p^{2}}{\partial \bar{y}})=\forall_{x}\frac{\partial (\bar{p}\bar{h})}{\partial \bar{x}}+\sigma \frac{\partial (\bar{p}\bar{h})}{\partial \bar{t}}$$

where $\forall_{x}=\frac{12\eta lU}{\left(1+6k_{n}^{\prime }\right)p_{0}h_{m}^{2}}$, $\sigma =\frac{24\eta lV}{(1+6k_{n}^{\prime })p_{0}h_{m}^{2}}$. Therefore the character of the velocity slip on the micro-scale is introduced to the Reynolds equation.

The load capacity of the aerostatic guideway *F* can be derived from the force balance according to the distribution of the pressure in gas pads:

(7) F=∫(p−pa)AdA

where *p_a_* is atmospheric pressure.

The stiffness of the aerostatic guideway is solved with the finite difference method:

(8) $$K=\frac{\partial F}{\partial h}$$

where *∂F* and *∂h* are the corresponding variation of load capacity and gas film thickness, respectively, from which the natural frequency of the aerostatic slides is deduced:

(9) $$f=1/2\pi \sqrt{K/m}=1/2\pi \sqrt{\partial F/(\partial h\cdot m)}$$

where *m* is the mass of the corresponding moving part, *i.e.*, the aerostatic slide.

**Figure 3.**
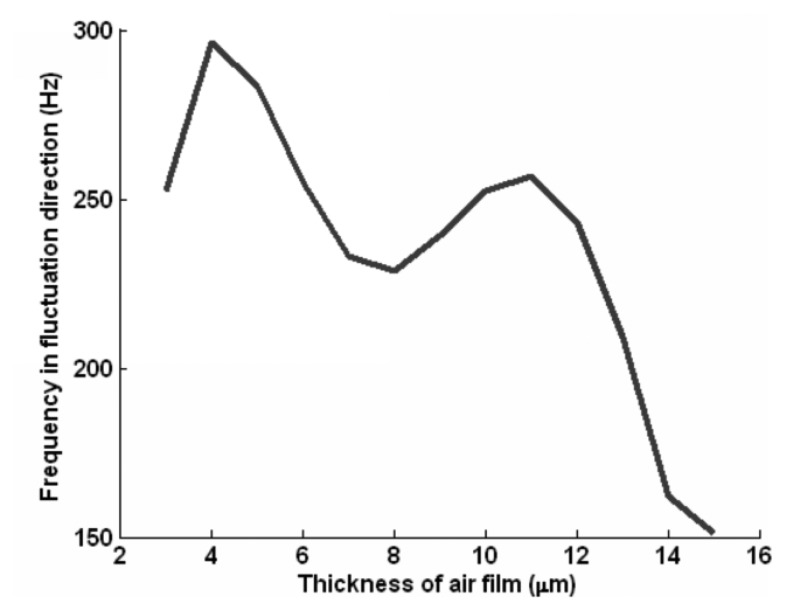
Frequency of the motion slider.

Figure 3 shows the fluctuation frequency of the motion slider. The vibration frequency of the motion slider is in the range of 150–300 Hz with the variation of gas film thickness.

### 2.4. Dynamic Model of Gas Fluctuations of Guideway

Self-excited oscillations are generated during machining due to the slight gas fluctuations of the aerostatic guideway. The air pressure will be increased when the air gap height decreases under the effect of applied loads. The aerostatic slider losses its equilibrium at a certain gap height as the gas film is compressible and it results in a periodical motion [19], which will generate motion perturbations at the tool-workpiece interface during the machining process. In order to analyze the perturbation phenomenon caused by the gas film fluctuation, the dynamic model of the motion slider of aerostatic guideway is established according to Figure 1. The motion of the slider is along the *x* direction, and the gas fluctuations along the *y* direction. In the simulation, the gas film between slider and guideway in Figure 1a is replaced by springs with a certain stiffness and damping. M, C, and K are the mass, damping and stiffness of the motion slider, respectively. The pneumatic self-induced oscillation of the moving slider will be caused by the pressure fluctuations of the gas film, and then the whole guideway system will vibrate. The coupling vibration equation is:

(10) $$M\ddot{y}+C\dot{y}+Ky=0$$

The vibration direction of the tool in the turning is very important for machining accuracy, this is mainly from the *y*(*t*) vibrations of the cross moving slide (in Figure 1a). The vibration amplitude reaches the peak when the resonance occurs. The vibration displacements y is given by:

(11) $$y(t)=Ae^{\alpha t}\sin(\omega_{d}+\varphi )$$

where $\alpha =\frac{c}{2M}$, *c* is the damping ratio, *ω_d_* the vibration frequency with damping, amplitude *A* and phase *φ* are:

(12) $$\{\begin{array}{c} A=\sqrt{{y_{0}}^{2}+{\left(\frac{{\dot{y}}_{0}+\alpha y_{0}}{\omega_{d}}\right)}^{2}}\\ \varphi =\text{arctg}\frac{y_{0}\omega_{d}}{{\dot{y}}_{0}+\alpha y_{0}} \end{array}$$

y_0_ and *ẏ*_0_ are the initial disturbance displacement and velocity, respectively.

## 3. Identifying Gas Fluctuations of Aerostatic Guideway

### 3.1. Measurement Equipment of the Fluctuation of the Aerostatic Slider

**Figure 4.**
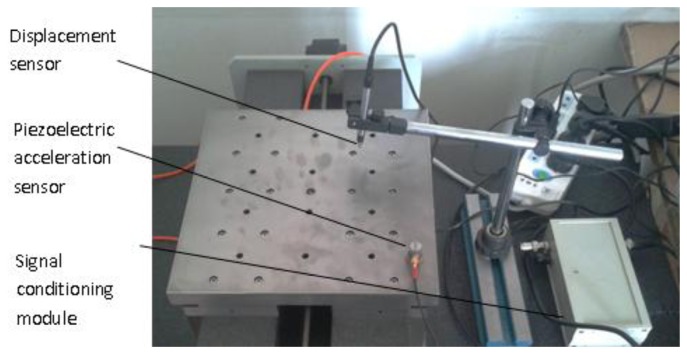
Measurement equipment of the gas fluctuation.

Gas film fluctuation is a self-excited oscillation, belonging to the class of vibrations with some frequency. A piezoelectric acceleration sensor (LC0101, Qinghuangdao Lance Test Technology Co. Ltd, Qinghuangdao, China) is used to measure the gas fluctuation of the aerostatic slider. Its sensitivity is 100 mV/g, range is 50 g, frequency range is 0.5–15,000 Hz, resolution 0.0002, resonant frequency 40 KHz. A signal conditioning box is used to acquire data. A capacitive displacement sensor (capaNCDT6300/6310, Germany meter iridium (Chinese) Micro-Epsilon Technology Co. Ltd, Beijing, China) is used to measure the displacement (linear range 10 mm, absolute error ≤ ±0.2%, resolution 0.001% (2 Hz), 0.01% (8 kHz), limit frequency 8 kHz (−3 dB)). The equipment is shown in Figure 4. Then, two groups of results are obtained, one measured before the working of the slider and upon the operation of air compressor, the other is when the gas film has been formed and the slider is working.

### 3.2. Processing of the Measured Signal with Power Spectral Density

Power spectral density is a statistical method, defined as the “power” (mean-square value) within the unit frequency band. It is the statistical result of the structure response which is excited by random dynamic loads and is a relationship curve with the values of power spectral density and frequency. In other words, the power spectrum is on the coordinate axis, frequency on the horizontal axis, and power on the vertical axis. Then the power spectral density is the amount changing with the frequency and distributed throughout the spectrum. It is a measurement for the mean square value of the random variable. *x*(*f*) and *S_x_*(*f*) are the functions describing the random signal *x*(*t*), *x*(*f*) is the amplitude of frequency spectrum, *S_x_*(*f*) is its power spectral density.

The random signal *x*(*t*), cannot be transferred by the Fourier transform and its spectrum cannot be obtained directly as it is not convergent and cannot meet the Dirichlet conditions. However, when the mean value of the random signal is zero, the correlation function of the signal can be convergent at *τ* → ∞, *i.e.*, *R_x_*(*τ* → ∞) = 0, thus it meeting the conditions of Fourier transform. According to the Fourier transform, the autocorrelation function *R_x_*(*τ*) is integral, and the power spectral density *S_x_(f)* is obtained:

(13) $$\int_{-\infty }^{\infty }S_{x}(f)df=\int_{-\infty }^{\infty }\underset{T\to \infty }{\lim}\frac{{\left|x(f)\right|}^{2}}{T}df=\underset{T\to \infty }{\lim}\int_{0}^{T}\frac{x^{2}(t)}{T}dt$$

**Figure 5.**
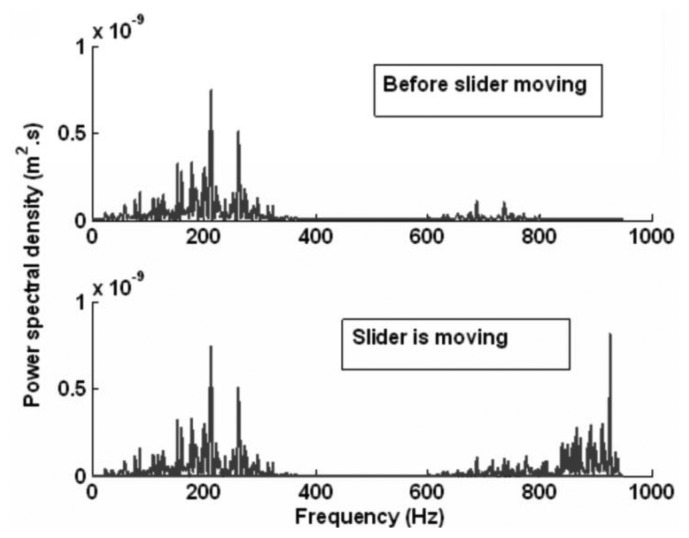
Measured result of the aerostatic guideway.

Figure 5 gives the power spectral density of the measured acceleration after signal processing. It shows that the frequency of the self-excited oscillation varies from 150 to 300 Hz, which is consistent with the result shown in Figure 3. When the slider moves, a new frequency of about 935 Hz appears, which is consistent with the modal result in Table 1. This is the natural frequency of the aerostatic guidway. This indicates that when the gas is pressured into the gas pads of the slider, a self-excited oscillation is generated, then the resonance vibration appears when the slider moves.

### 3.3. Spectral Signs of Unbalance of Guideway Parts

The guideway system will reciprocate around the equilibrium point with some interference. In the time domain, the vibration signal is a series of impulse waveforms. In the frequency domain, it includes the corresponding frequency of the guideway system. When the aerostatic guideway is working, a harmonic vibration may be caused by the fluctuation of gas film between the slide and guideway [20]. In the research, the calculated frequency of the slider is in the range of 150–300 Hz, and the natural frequency of the guideway system is 932.38 Hz. The measured signal of the aerostatic guideway system includes the motion frequency of the slider, and also includes the natural frequency of the guideway system. With the simulation and experiment results, this paper indicates the spectrum signs of the unbalance of guidway parts, and validates the derived conclusions.

## 4. Results and Discussion

The purpose of the study is to extract the main features and to identify the gas fluctuations caused by the unbalance of aerostatic guideways. Through the dynamic model of the motion sliders, the corresponding frequency of the whole guideway system is obtained. The power spectral density is used to process the measured signal in the frequency domain, and the frequency is shown in Figure 5. The main feature is extracted from the component frequency of the aerostatic guideway system. By comparing with the two results in Figure 5, we find that the range of the frequency before the slider moves is from 150 to 300 Hz, which is consistent with the calculated result in Figure 3. When the slider moves, the newly appearing frequency at 935 Hz is consistent with the modal result of 932.68 Hz in Table 1. This is the natural frequency of the aerostatic guidways, which indicates that when the gas is pressured into the gas pads of the slider, a self-excited oscillation is generated and as a result, the unbalance of the aerostatic guideway occurs when the slider moves.

According to the results, the velocity slip method reflects the effect of the rarefied air. Therefore, Equation (6) is very effective to simulate the state of gas flow in the slider, dynamic model and frequency distribution range can be derived with the Equation (6). Combined with the main features of the guideways in Table 1, the unbalance of the aerostatic guideway caused by the gas fluctuation is identified from the experimental results with the power spectral density.

## 5. Conclusions

The gas flow state is analyzed using the slip velocity method. The dynamic model of the motion slider is established according to the natural frequency. The fluctuation frequency of the gas film of the whole sliders in the *y* direction (turning direction) in Figure 1a with gas thickness has been derived. Power spectral density is used to process the measured signal of the aerostatic guideway. The frequency characteristics of the unbalance of aerostatic guideway caused by gas fluctuation of aerostatic guideway are extracted from the measured signal, and the basic frequency of the guideway system is diagnosed according to the spectral characteristics. It agrees with the results calculated by the motion equations and the finite element method.

## Figures and Tables

**Table 1.** Fifth order natural frequencies of guideway system.

**Order Number**	**1**	**2**	**3**	**4**	**5**
Theory (Hz)	932.68	2,570.8	5,040.1	8,331.6	12,446
ANSYS (Hz)	932.68	2,571	5,040	8,330	12,440
